# A Rare Case of Plasma Cell Granuloma

**DOI:** 10.1155/2020/8861918

**Published:** 2020-12-26

**Authors:** Archana P. Kanteti, W. Patrick Kelsey, Ernesto Martinez Duarte

**Affiliations:** ^1^Department of Pathology and Microbiology, University of Nebraska Medical Center, USA; ^2^Creighton University School of Dentistry, USA

## Abstract

Plasma cell granulomas (PCGs) or inflammatory pseudotumors are nonneoplastic lesions that consist of predominantly antibody-secreting plasma cells and innate immune cells such as neutrophils, macrophages, and eosinophils. Unlike in multiple myeloma, the plasma cells are polyclonal and present in a spindly fibroblast-rich stromal background. These lesions mainly occur in the lungs; however, they can arise in other organs. PCGs from the gingiva are extremely rare, and a proper diagnosis is crucial to treat these patients further. These tumors have an increased number of plasma cells that are immunoreactive with CD138 and are polyclonal for kappa and lambda light chains, confirming these proliferations' nonneoplastic nature. Surgical resection with clear margins, when possible, is the primary choice of treatment. Radiation and anti-inflammatory steroid therapy are other therapeutic approaches. Critical and careful examination by a pathologist is necessary to rule out plasma cell neoplasms. Here, we report a rare occurrence of gingival PCG in an elderly male.

## 1. Introduction

Plasma cell granulomas (PCGs) are benign inflammatory growths that commonly occur in the lungs and are rarely found in the oral cavity [1, 2]. They typically consist of a mass collection of polyclonal plasma cells [2]. In the oral cavity, plasma cell granuloma occurring in the gingiva is rare [2]. Mass-forming gross disease can mimic a malignant process. Accurate diagnosis is based on gross, radiographic, morphologic, and immunophenotypic characteristics [3]. We present and discuss the underlying pathology in the general context of PCGs.

## 2. Case History

**Figure 1:**
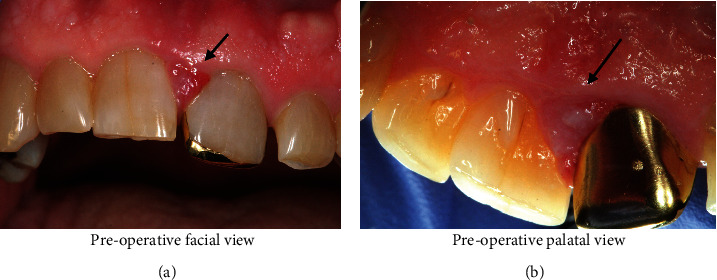


**Figure 2:**
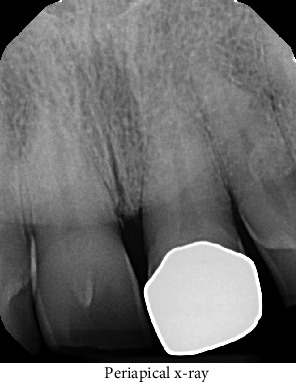


The patient is a 71-year-old male with a past medical history significant for prediabetes, hyperlipidemia, and gastroesophageal reflux disease. He presented for a periodontal consultation due to an inflamed gingival swelling in the #8-9 interproximal space. The patient reported minimal to no discomfort but was aware that the site bled profusely when he flossed. He believed he was able to remove a fragment of debris from the area, which temporarily improved the swelling, but over time, the swelling and bleeding did not completely resolve. The patient does brush and floss regularly and did not note any recent trauma. Upon physical examination, the soft tissues between teeth #8-9 were erythematous. The lesional tissue had a sessile base and was soft but not fluctuant to pressure (Figure 1). The lesion was 5 mm wide and 6 mm tall and extended through the interdental papilla space. There was an open contact between the gold onlay restoration at #9 and the tooth at #8. The lesion appeared to be a reactive process due to irritation, possibly from the open contact, such as a pyogenic granuloma. Periapical radiographs showed 1-2 mm of horizontal bone loss between teeth #8-9 (Figure 2). The patient was advised to use 0.12% chlorhexidine gluconate rinse three times per day for thirty-second intervals, focus on interproximal plaque removal for three weeks, and then return for follow-up. After three weeks of home treatment, minimal improvement of the gingival lesion was noted. The patient underwent an excisional biopsy with complete removal and degranulation of the papillary tissues. The dentist recommended using salt water rinses, to reduce plaque formation in the interproximal space.

**Figure 3:**
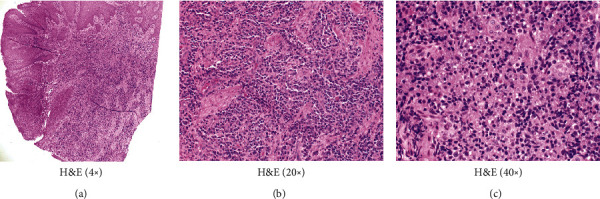


Microscopic examination by H&E showed benign squamous epithelium with underlying submucosa filled with sheets of numerous bland-appearing plasma cells (Figure 3). The plasma cells have a characteristic eccentrically placed nucleus with a nuclear Hoff (Figure 3(b)). Lymphocytes and histiocytes are also present infiltrating the stroma. Characteristic Russel bodies are present in the cytoplasm of the plasma cells (Figure 3(c)).

**Figure 4:**
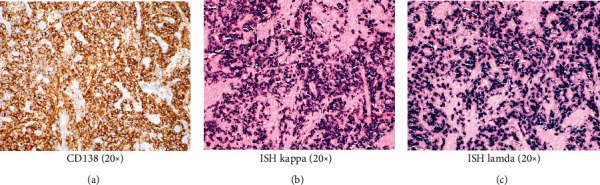


Immunohistochemical staining for CD138, a plasma cell marker, was diffusely positive in the lesional tissue highlighting the abundance of plasma cells (Figure 4(a)). Kappa and lambda light chain expression by in situ hybridization showed polytypic plasma cells (Figures 4(b) and 4(c)). The following findings were all consistent with a diagnosis of plasma cell granuloma.

**Figure 5:**
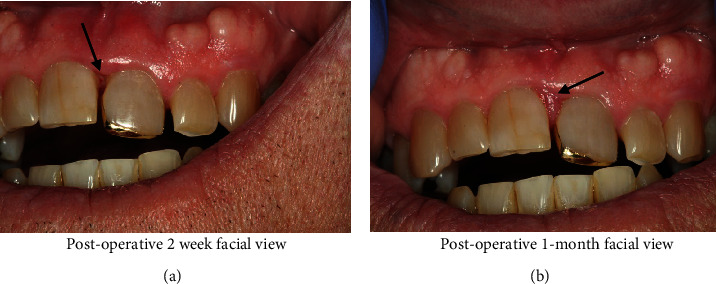


The patient returned for postoperative follow-up at two weeks and eight weeks (Figure 5). Over two weeks (Figure 5(a)), the soft tissues between teeth #8 and #9 began to granulate via secondary intention. By eight weeks (Figure 5(b)), a more normal-appearing, much thinner, interdental papilla was visible with some residual inflammation. Probing with a UNC 15 mm probe revealed resistance at 4 mm deep with minimal bleeding. The patient was instructed to return to his general dentist for regular recall prophylaxis and exam appointments and referral request if the lesion recurred.

## 3. Discussion

Plasma cell granuloma (PCG) is also known as “inflammatory pseudotumor” [4]. It is an infrequent nonneoplastic lesion of uncertain etiology [5]. Zoon first recognized it in 1952 as balanitis plasmacellularis, or plasma cell balanitis [6]. The first use of the term PCG occurred in 1950 and 1960. It was first reported as an entity in the gingiva in 1968 [7, 8] and described as an inflammatory pseudotumor in 1973 by Bahadori and Liebow [7]. It is also known as inflammatory myofibrohistiocytic proliferation, inflammatory myofibroblastic tumor, and xanthomatous pseudotumor [8, 9].

The pathogenesis of PCG is still unclear. It is thought to be reactive and associated with periodontitis, periradicular inflammation due to a foreign body's presence, or the presence of a foreign antigen such as EBV and HHV8 [10–12]. Amlodipine and cyclosporines are known causative agents for plasma cell granulomas [10, 13, 14].

PCG most commonly arises from the lungs, although it can present in various other organs in the body [8, 15]. In the head and neck regions, it accounts for fewer than 5% of all extrapulmonary cases [10, 16]. The orbit is the most common location, followed by the meninges, paranasal sinuses, infratemporal fossa, and soft tissues [16, 17]. The temporal bone, skull base, and facial nerve are rarely involved [10].

It also involves the oral cavity, with the gingiva being the most commonly affected area with an equal rate of involvement of the maxillary and mandibular gingiva [18, 19]. Early lesions of nonneoplastic plasma cell infiltrate of the oral cavity were called atypical gingivostomatitis, idiopathic gingivostomatitis, and allergic gingivostomatitis [19]. They were considered secondary to chewing gum, dentifrices, or foreign oral substances [19].

While relatively benign, these tumors may simulate malignancy, become symptomatic secondary to size or location, and present a diagnostic challenge to clinicians, pathologists, and radiologists [5]. Reports of recurrences are few, hence the importance of thinking about the diagnosis and treating it properly [20, 21].

PCG does not have an age preference [22]. Even though they can present as multiple simultaneous lesions, they are most commonly solitary [22]. More often than not, these lesions are asymptomatic and detected incidentally during routine dental evaluations [10, 15]. The alveolar mucosa is not usually involved, and subsequent bone loss depends on the lesion's etiology and chronicity. The patient can complain of bleeding and an inability to maintain oral hygiene [15, 23].

Microscopically, they are composed of a mixture of inflammatory cells, predominantly a polyclonal population of plasma cells, admixed with neutrophils and lymphocytes [18, 23]. The plasma cells show the classic eccentric nuclei, with clear, perinuclear Hoffs and numerous cytoplasmic Russell-Fuchs bodies [19]. The stroma tends to be fibrotic with areas of spindled, reactive fibroblastic proliferation [2, 5]. Russell bodies are large, eosinophilic, homogeneous immunoglobulin-containing inclusions in the plasma cell [8]. The presence of these bodies further supports the reactive nature of the plasma cell infiltrates [15].

Immunohistochemistry studies show that the plasma cells are positive for CD138 [24, 25]. The plasma cells are polyclonal illustrated by positive reactivity for both kappa and lambda light chains by immunohistochemistry or in situ hybridization [24, 25].

Treatment of the lesion depends on the size and associated symptomatology. It includes scaling, excisional biopsy, or possible extraction of the adjacent tooth if involved [26]. A few cases have reported the use of steroid therapy and spontaneous regression [27–29]. Due to these tumors' unencapsulated nature, surgical excision with wide margins is necessary to prevent recurrence [10, 26, 30].

The differential diagnosis of plasma cell infiltrates in the oral cavity includes plasma cell mucositis, plasma cell gingivitis, multiple myeloma, solitary myeloma, and plasmacytomas [2, 31]. Multiple myeloma and solitary myeloma are frequently solitary bone tumors, whereas plasmacytoma and plasma cell granulomas primarily affect the soft tissues [18, 32]. Differentiating the type of soft tissue tumor is crucial, as plasma cell granuloma is a benign growth, whereas plasmacytoma can represent the early stages of multiple myeloma [32].

Morphology and basic immunostains are enough to differentiate plasma cell mucositis, gingivitis, and plasmacytomas. Plasma cell granuloma will show a plasma cell-rich inflammatory infiltrate composed of polyclonal plasma cells [33].

Other rare entities that can affect the oral cavity are oral fibroma, peripheral giant cell reparative granulomas, and pyogenic granulomas. All these lesions show a fibrous or vascular rich stroma instead of a plasma cell predominant infiltrate [3].

## References

[1] Agrons G. A., Rosado-de-Christenson M. L., Kirejczyk W. M., Conran R. M., Stocker J. T. (1998). Pulmonary inflammatory pseudotumor: radiologic features. *Radiology*.

[2] Acevedo A., Buhler J. E. (1977). Plasma-cell granuloma of the gingiva. *Oral Surgery, Oral Medicine, and Oral Pathology*.

[3] Andrade D. M. S., Martins S. J., Paz O., Cardozo J. B., Novaes A. E., Santiago M. B. (2006). Inflammatory pseudotumor: a diagnostic dilemma. *European Journal of Internal Medicine*.

[4] Batich K. A., William St Clair E., McLendon R. E., Sampson J. H. (2013). Complete response to steroids in dural inflammatory pseudotumor associated with Still's disease. *Journal of Clinical Neuroscience*.

[5] Forcucci J., Butler-Williams S., Miller N., Lazarchick J. (2015). Plasma cell granuloma: an entity within the spectrum of IgG4-related disease. *Annals of Clinical and Laboratory Science*.

[6] Zoon J. J. (1952). Balanoposthite chronique circonscrite bénigne à plasmocytes. *Dermatologica*.

[7] Bahadori M., Liebow A. A. (1973). Plasma cell granulomas of the lung. *Cancer*.

[8] Ide F., Shimoyama T., Horie N. (2000). Plasma cell granuloma of the oral mucosa with angiokeratomatous features: a possible analogue of cutaneous angioplasmocellular hyperplasia. *Oral Surgery, Oral Medicine, Oral Pathology, Oral Radiology, and Endodontics*.

[9] Vahedi A., Moya-Plana A., Guyot S., Touré G. (2018). Plasma cell granuloma of the jaw and the infratemporal fossa: a clinical case. *Journal of Oral and Maxillofacial Surgery*.

[10] Ajibade D. V., Tanaka I. K., Paghda K. V., Mirani N., Lee H. J., Jyung R. W. (2010). Inflammatory pseudotumor (plasma cell granuloma) of the temporal bone. *Ear, Nose, & Throat Journal*.

[11] Fukunaga A., YOSHIDA K., OTANI M. (1998). Plasma cell granuloma extending from the extracranial to the intracranial space associated with Epstein-Barr virus infection. *Neurologia Medico-Chirurgica (Tokyo)*.

[12] Arber D. A., Kamel O. W., van de Rijn M. (1995). Frequent presence of the Epstein-Barr virus in inflammatory pseudotumor. *Human Pathology*.

[13] Vishnudas B., Sameer Z., Shriram B., Rekha K. (2014). Amlodipine induced plasma cell granuloma of the gingiva: a novel case report. *Journal of Natural Science, Biology and Medicine*.

[14] Kim S. S., Eom D. W., Huh J. R. (2002). Plasma cell granuloma in cyclosporine-induced gingival overgrowth: a report of two cases with immunohistochemical positivity of interleukin-6 and phospholipase C-gamma1. *Journal of Korean Medical Science*.

[15] Akdogan N., Yalçın B., Aksoy G. G., Tuna E. E., Ünal D. T. (2017). A case of plasma cell granuloma located on the gingiva. *The American Journal of Dermatopathology*.

[16] Ballesteros E., Osborne B. M., Matsushima A. Y. (1998). Plasma cell granuloma of the oral cavity: a report of two cases and review of the literature. *Modern Pathology*.

[17] Kilinc M., Ertürk İ. Ö., Uysal H., Birler K., Evrenkaya T., Akkalyoncu B. B. (2002). Multiple plasma cell granuloma of the central nervous system: a unique case with brain and spinal cord involvement. Case report and review of literature. *Spinal Cord*.

[18] Laco J., Kamarádová K., Mottl R. (2015). Plasma cell granuloma of the oral cavity: a mucosal manifestation of immunoglobulin G4-related disease or a mimic?. *Virchows Archiv*.

[19] Manohar B., Bhuvaneshwari S. (2011). Plasma cell granuloma of gingiva. *Journal of Indian Society of Periodontology*.

[20] Balsarkar D. J., Ranadive N. U., Gore M. A., Joshi M. A. (2002). Recurrent inflammatory pseudotumor of small bowel mesentery presenting as perforative peritonitis. *Indian Journal of Gastroenterology*.

[21] Garcia B. A., Tinsley S., Schellenberger T., Bobustuc G. C. (2012). Recurrent inflammatory pseudotumor of the jaw with perineural intracranial invasion demonstrating sustained response to rituximab. *Medical Oncology*.

[22] Brage-Varela A., Boullosa P. E., Otero J. P., Rodríguez M. B., Novoa M. L., Monreal F. A. (2010). Multifocal hepatic inflammatory pseudotumor: spontaneous regression in a diabetic patient. *Revista Española de Enfermedades Digestivas*.

[23] Jeyaraj P., Bandyopadhyay T. K., Naresh N., Sahoo N. K. (2015). Value of immunohistochemistry in diagnosing a rare case of maxillofacial plasma cell granuloma masquerading as a gingival epulis. *Journal of Maxillofacial and Oral Surgery*.

[24] Buccoliero A. M., Caldarella A., Santucci M. (2003). Plasma cell granuloma--an enigmatic lesion: description of an extensive intracranial case and review of the literature. *Archives of Pathology & Laboratory Medicine*.

[25] Deb J., Chaudhuri A. D., Saha R., Chakraborty S. (2007). Plasma cell granuloma of lung. *Journal of the Indian Medical Association*.

[26] Piroth L., Menecier P., Charvillat L., Naouri A., Kisterman J. P. (1996). Diagnosis and treatment of pulmonary plasma cell granuloma. A propos of a case successfully treated by antibiotics. *La Revue de Médecine Interne*.

[27] Biecker E., Zimmermann A., Dufour J. F. (2003). Spontaneous regression of an inflammatory pseudotumor of the liver. *Zeitschrift für Gastroenterologie*.

[28] Brown K. C., McCarthy V. P., Gaines T. (1998). Spontaneous resolution of a plasma cell granuloma in a 9-year-old. *Journal of the Association for Academic Minority Physicians*.

[29] Bush A., Sheppard M. N., Wahn U., Warner J. O. (1992). Spontaneous arrest of growth of a plasma cell granuloma. *Respiratory Medicine*.

[30] Chen C. H., Huang W. C., Liu H. C., Chen C. H., Chen T. Y. (2008). Surgical outcome of inflammatory pseudotumor in the lung. *The Thoracic and Cardiovascular Surgeon*.

[31] Pandav A. B., Gosavi A. V., Lanjewar D. N., Jagadale R. V. (2012). Gingival plasma cell granuloma. *Journal of Dental Research*.

[32] Rajkumar S. V., Kumar S. (2016). Multiple myeloma: diagnosis and treatment. *Mayo Clinic Proceedings*.

[33] Solomon L. W., Wein R. O., Rosenwald I., Laver N. (2008). Plasma cell mucositis of the oral cavity: report of a case and review of the literature. *Oral Surgery, Oral Medicine, Oral Pathology, Oral Radiology, and Endodontics*.

